# The Diagnostic Value of Soluble Triggering Receptor Expressed on Myeloid Cells for Patients with Acute Stone Pyelonephritis

**DOI:** 10.3390/diagnostics14070777

**Published:** 2024-04-07

**Authors:** Metin Özsoy, Miraç Ataman, Serhat Kazım Şahin, İbrahim Şenocak, Artuner Varlibaş, Ercan Yuvanç, Aydın Çifci, Mustafa Kemal Başaralı, Gül Kırtıl, Erdal Yilmaz

**Affiliations:** 1 Department of Infectious Diseases and Clinical Microbiology, Health Sciences University, Ankara Training and Research Hospital, 06050 Ankara, Turkey; 2 Department Urology, Kırıkkale University Faculty of Medicine, 71300 Kırıkkale, Turkey; 88m.ataman@gmail.com (M.A.); srhtkzm@hotmail.com (S.K.Ş.); ibrahimsenocak@kku.edu.tr (İ.Ş.); ercanyuvanc@gmail.com (E.Y.); erdaly69@mynet.com (E.Y.); 3 Department Internal Medicine, Kırıkkale University Faculty of Medicine, 71300 Kırıkkale, Turkey; artunervarlibas@gmail.com (A.V.); dr.aydin.71@hotmail.com (A.Ç.); 4 Department Medical Biochemistry, Republic of Turkey Ministry of Health, General Directorate of Public Health, 06800 Ankara, Turkey; kemalbasarali@yahoo.com; 5 Department of Medical Biochemistry, Health Sciences University, Ankara Training and Research Hospital, 06230 Ankara, Turkey; gulkirtil@hotmail.com

**Keywords:** biomarkers, diagnosis, pyelonephritis, soluble triggering receptor expressed on myeloid cells

## Abstract

Soluble triggering receptor expressed on myeloid cells (sTREM-1) is a new biomarker that can be used for the diagnosis and monitoring of urinary system infections. This study aimed to evaluate the diagnostic performance of serum sTREM-1 in patients with a diagnosis of acute stone pyelonephritis (ASP). This prospective study included 46 patients with a diagnosis of ASP and a control group of 23 individuals without urinary system infection. Blood samples were taken from participants upon hospital admission, and basal serum sTREM-1 levels were analyzed using the ELISA method. Serum sTREM-1 concentrations were measured after treatment of ASP patients. Basal leukocyte counts, C-reactive protein (CRP) levels, procalcitonin (PCT), and sTREM-1 (98.6 vs. 68.4 pg/mL, *p* < 0.001) levels were higher in the ASP group compared to the control group. After treatment, the median leukocyte counts, PCT, and sTREM-1 levels decreased and were similar to those of the control group. The median CRP level also decreased after treatment, but it remained higher than that of the control group. In predicting patients with ASP, the baseline sTREM-1 exhibited a sensitivity of 74.6% and a specificity of 78.2%, while its diagnostic performance was lower than that of leukocyte counts, CRP, and PCT. Despite the findings that levels of sTREM-1 were higher upon hospital admission in patients with ASP and significantly decreased after treatment, the utility of sTREM-1 as a biomarker for predicting patients with ASP remains constrained when compared to established inflammatory markers.

## 1. Introduction

Pyelonephritis, which is an infection of the upper urinary tract, can lead to lasting damage in the upper urinary system if not addressed immediately [1]. In patients with pyelonephritis, leukocytosis, C-reactive protein (CRP), and procalcitonin (PCT) are the most commonly used biomarkers for diagnosis and treatment monitoring [2,3,4].

Soluble triggering receptor expressed on myeloid cells-1 (sTREM-1) is a member of the immunoglobulin family, is expressed on neutrophils and monocytes, and was first identified in 2000 [5]. While sTREM-1 levels show an increase in inflammation due to bacterial and fungal pathogens, they may exhibit a weak increase in cases of non-infectious inflammatory events [6,7]. sTREM-1 is shed from the cell surface and released into body fluids, including plasma, pleural effusion, sputum, and urine, during the proteolytic cleavage process triggered by metalloproteinases that are activated by lipopolysaccharides [8]. Increased levels of sTREM-1 in body fluids during an infection can be detected using serological assays such as the ELISA test, enabling differentiation between infectious and non-infectious inflammation [5,9]. Previous studies on sepsis, pneumonia, acute respiratory distress syndrome, and urinary tract infections (UTIs) have indicated that sTREM-1 could serve as a potential biomarker [10,11,12,13,14].

Given the observed rise in sTREM-1 concentrations during inflammatory conditions, we hypothesized that individuals with acute stone pyelonephritis (ASP) might exhibit elevated sTREM-1 concentrations relative to patients without UTIs and that there might be a notable decrease in sTREM-1 levels in ASP patients after treatment. Therefore, the objectives of this study were to investigate the variance in sTREM-1 concentrations between patients diagnosed with ASP and patients without UTIs, to assess the diagnostic efficacy of sTREM-1 in identifying ASP patients, and to examine the post-treatment alterations in sTREM-1 concentrations.

## 2. Materials and Methods

This prospective study was conducted with patients being followed for a diagnosis of ASP at the Kirikkale University Faculty of Medicine Urology Outpatient Clinic between June 2022 and May 2023, as well as adult patients without UTIs. The study received approval from the Ethics Committee of the Kirikkale University Faculty of Medicine (approval date: 3 August 2023; number: 11/02) and adhered to the ethical regulations and principles stipulated in the Declaration of Helsinki. Informed consent was obtained from all participants.

The inclusion criteria for the patients with ASP included being aged between 18 and 90 years, having a preliminary diagnosis of ASP, and signing the consent form for participation. Patients under the age of 18 and over 90, patients with underlying malignancies (such as prostate or bladder cancer, etc.), patients taking immunosuppressive drugs, and those receiving chemotherapy or radiotherapy were excluded from the study.

The control group was selected from among outpatients who had no history or symptoms of UTIs. The following inclusion criteria were applied: individuals aged between 18 and 90 years who presented to the outpatient clinic for reasons other than ASP or any UTI and who had blood taken for routine biochemical tests.

### 2.1. Study Protocol

Clinical, demographic, laboratory, and radiological findings were recorded promptly in the patients’ files during follow-up. Clinically, patients who had a fever, dysuria, pollakiuria, costovertebral tenderness, radiological imaging results consistent with pyelonephritis, and significant elevations in leukocyte count, CRP, and PCT levels were evaluated as having pyelonephritis [15]. Biochemical parameters were analyzed using venous blood samples collected during outpatient evaluations after a 12 h fasting period. After treatment, blood samples were collected again from the patients with ASP. All samples were analyzed in a single laboratory using the same methodology as described below.

### 2.2. Biochemical Analysis

Blood samples were obtained from the antecubital vein at presentation for the evaluation of inflammatory parameters. The samples were allowed to coagulate at room temperature for 10–20 min and then centrifuged at 3000× *g* at 4 °C for 10 min and stored at −80 °C. Complete blood cell counts and biochemical parameters were measured with a Beckman Coulter LH 780 device (Mervue, Galway, Ireland) and a Hitachi Modular P800 autoanalyzer (Roche Diagnostics Corp., Indianapolis, IN, USA). Levels of leukocytes (optical laser scattering method) and CRP (immunoturbidimetric method) were measured.

Concentrations of human sTREM-1 in the serum samples were measured using the enzyme-linked immunosorbent assay (ELISA) method, which is based on the principle of the double antibody sandwich technique, following the manufacturer’s instructions (catalog no: 201-12-0311, SunRed Biotechnology Co., Ltd., Shanghai, China). In this kit, the micro-ELISA plate is coated with an antibody specific to human sTREM-1. Standards were prepared by adding 50 µL of each reference solution to the designated micro-ELISA plate wells. For the test samples, 40 + 10 µL of sTREM-1-specific antibody was added to the micro-ELISA plate wells. Subsequently, 50 µL of streptavidin-horseradish peroxidase (HRP) conjugate was applied to each well to enable specific binding with the sTREM-1 antibodies. The plate was incubated at 37 °C for 60 min to ensure optimal antibody–antigen interaction. Following incubation, the contents of each well were aspirated (BioTek ELx50, BioTek Instruments, Winooski, VT, USA), and the wells were washed five times. After washing, 50 µL of Chromogen Solution A and 50 µL of Chromogen Solution B were added to each well. The plate was then incubated in the dark at 37 °C for 10 min, during which time the wells turned blue, indicating the presence of enzyme–substrate reactions. The reaction was halted by adding 50 µL of Sulfuric Acid Stop Solution to each well, causing the solution to change from blue to yellow. The optical density (OD) was measured spectrophotometrically at a wavelength of 450 ± 2 nm using a microplate reader (BioTek ELx800, BioTek Instruments, Winooski, VT, USA). These OD values are directly proportional to the concentration of human sTREM-1. Human sTREM-1 concentrations were determined by comparing the OD values of the samples to a standard curve. The minimum measurable amount of human sTREM-1 was 3.102 pg/mL. The inter-assay and intra-assay variations were recorded as less than 12% and 10%, respectively [16].

### 2.3. Statistical Analysis

All data were analyzed with IBM SPSS Statistics for Windows 20.0 (IBM Corp., Armonk, NY, USA). Numerical data determined to be normally distributed based on the results of Kolmogorov–Smirnov tests are given as mean ± standard deviation (SD) values, while non-normally distributed variables are given as median (25th–75th quartile) values. For comparisons between groups, Student *t*-test and Mann–Whitney U test were used in line with the normality of the considered distribution. Categorical variables are given as numbers and percentages, and inter-group comparisons were conducted with Chi-square and Fisher exact tests. Spearman correlation analyses were applied to evaluate the relationships between numerical variables. Receiver operating characteristic (ROC) curve analysis was applied to assess diagnostic performance. Threshold values were determined by the Youden index method. Comparison of the areas under the curves (AUCs) was performed with a nonparametric approach using the theory on generalized U-statistics to generate an estimated covariance matrix, as previously reported by DeLong et al. [17]. Significance was accepted at *p* < 0.05 (*) for all statistical analyses.

## 3. Results

In the study, 46 ASP patients were analyzed, comprising 27 males and 19 females with a mean age of 47.7 ± 16.8 years, along with a control group of 23 individuals with a mean age of 48.8 ± 12.7 years. The mean age, gender distribution, and distribution of additional diseases were similar between the ASP group and the control group. Among the patients with ASP, all had pyuria, 63% had positive nitrites, 91.3% had positive leukocyte esterase, and 60.9% had a positive urine culture (Table 1).

**Figure 1:**
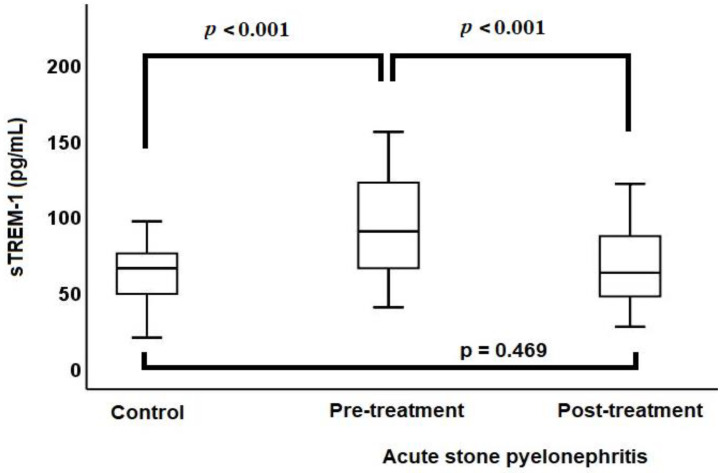
Comparison of sTREM-1 levels between acute stone pyelonephritis and control groups via a box-plot chart.

The median baseline sTREM-1 level (98.6 vs. 68.4 pg/mL, *p* < 0.001) (Figure 1), median baseline leukocyte count (12.4 vs. 7.2 × 10^3^/µL, *p* < 0.001), median baseline CRP level (129.7 vs. 1.5 mg/L, *p* < 0.001), and median baseline PCT level (0.26 vs. 0.03 µg/L, *p* < 0.001) were higher in the ASP group compared to the control group.

ASP patients were treated with antibiotics for a period ranging from 7 to 14 days, with the median duration being 7 days. After treatment, there was a reduction in leukocyte counts (12.4 vs. 7.6 × 10^3^/µL, *p* < 0.001), CRP levels (129.7 vs. 4.5 mg/L, *p* < 0.001), PCT levels (0.26 vs. 0.04 µg/L, *p* < 0.001), and sTREM-1 levels (98.6 vs. 65.7 pg/mL, *p* < 0.001). Following the treatment of ASP patients, there were no significant differences in the leukocyte counts, PCT levels, and sTREM-1 levels (Figure 1) when compared with the control group. However, after treatment, the CRP levels of ASP patients remained high compared to the control group (4.5 vs. 1.5 mg/L, *p* < 0.001) (Table 2).

In the control group, sTREM-1 levels were not correlated with leukocyte counts, CRP, or PCT levels. In the ASP group, prior to treatment, there was a positive correlation of sTREM-1 levels with leukocyte counts (r = 0.325, *p* = 0.028), CRP levels (r = 0.412, *p* = 0.004), and PCT levels (r = 0.535, *p* < 0.001). The post-treatment changes in sTREM-1 levels only showed a positive correlation with changes in PCT levels (r = 0.311, *p* = 0.036) (Table 3).

**Figure 2:**
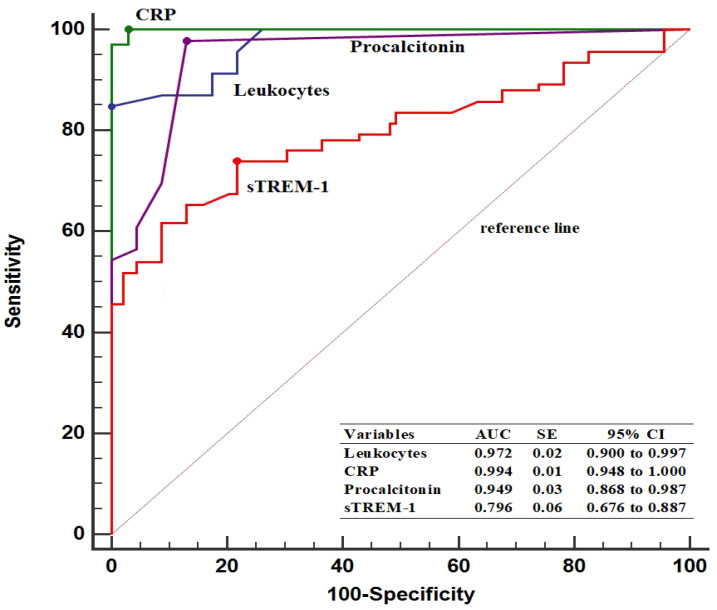
Diagnostic performance of inflammatory parameters in predicting patients with acute stone pyelonephritis. AUC, area under the curve; CI, confidence interval; CRP, C-reactive protein; SE, standard error; sTREM-1, soluble triggering receptor expressed on myeloid cells.

In predicting patients with ASP, the baseline sTREM-1 threshold value was determined to be >75.4 pg/mL, with 74.6% sensitivity and 78.2% specificity. Furthermore, pre-treatment sTREM-1 levels had a lower AUC value compared to leukocyte counts (0.796 vs. 0.972, *p* < 0.001), CRP levels (0.796 vs. 0.994, *p* < 0.001), and PCT levels (0.796 vs. 0.949, *p* < 0.001) (Figure 2).

## 4. Discussion

To the best of our knowledge, this study is one of few studies to date assessing sTREM-1 in patients with ASP. Patients with ASP exhibited higher levels of sTREM-1 compared to the control group. Additionally, the sTREM-1 levels of ASP patients were significantly decreased after treatment, showing similarity to the levels measured in the control group. The results of this study indicated that sTREM-1 exhibited acceptable diagnostic performance in predicting patients with ASP.

While current biomarkers like CRP, PCT, and erythrocyte sedimentation rate are extensively utilized in diagnosing and monitoring pyelonephritis, their effectiveness remains debated due to variable sensitivity and specificity values [18]. Furthermore, ultrasonography findings are normal in the majority of uncomplicated cases [19,20]. In a previous study conducted with children diagnosed with acute pyelonephritis and lower UTIs, the diagnostic values of serum PCT and interleukin (IL)-1β levels were compared with sedimentation rate and CRP levels [2]. In children diagnosed with acute pyelonephritis, these inflammatory parameters were reported to be higher compared to children with lower UTIs. Additionally, in predicting acute pyelonephritis, the sensitivities and specificities were shown to be 31.0% and 84.7% and 27.2% and 90.0% for serum PCT and IL-1β, respectively. On the other hand, it was also demonstrated that they had lower diagnostic performance compared to the markers of sedimentation rate and CRP [2]. In another study assessing levels of copeptin, CRP, PCT, and IL-6 for distinguishing between lower and upper UTIs, it was reported that all biomarker levels were higher than those in the control group before antibiotic treatment, and after 7 days of antibiotic treatment, all biomarker levels had decreased in parallel with clinical response [3]. It was also reported that copeptin levels had a lower diagnostic performance in distinguishing between lower and upper UTIs compared to other biomarkers [3]. These findings suggest that different inflammatory parameters may have varying diagnostic performances in UTIs. Therefore, new biomarkers are required to predict UTIs, including pyelonephritis.

Circulating sTREM-1 levels have been found to rise in cases of various inflammatory diseases [10,11,12,13,14]. TREM-1 is associated with transmembrane adapter molecule-12 (DAP12). Therefore, TREM-1 mediates neutrophil and monocyte activation and plays an important role in inflammatory reactions [5]. It has been indicated that the expression of TREM-1 on neutrophils can induce cytokines in cases of sepsis caused by *Escherichia coli.* In bacterial sepsis, the TREM-1 pathway on neutrophils may be a key player in inducing an adequate inflammatory response and establishing a bactericidal response [9]. A previous study reported that levels of sTREM-1 in urine are more sensitive than levels of leukocytes, CRP, and PCT for the early diagnosis of sepsis and for dynamically evaluating its severity and prognosis [21]. In a study conducted with patients with acute cholangitis, it was demonstrated that sTREM-1 levels exhibited a sensitivity of 58.6% and a specificity of 86.1% in distinguishing patients with sepsis from those without sepsis. Additionally, compared to a control group without acute cholangitis or other infections, sTREM-1 levels showed a sensitivity of 93.1% and a specificity of 90.8%. However, the authors reported that sTREM-1 levels were a better marker than CRP or PCT for monitoring patient responses to antimicrobial therapy and biliary drainage [22]. It has also been suggested that sTREM-1 could be a more sensitive and specific biomarker than traditional indicators, namely CRP and PCT levels, in diagnosing infectious diseases and predicting their prognosis [23]. In a meta-analysis by Jiyong et al., the diagnostic precision of sTREM-1 in determining bacterial infections was assessed [24]. The criteria for incorporating studies into their review included the use of recognized testing methods for sTREM-1. In total, the review encompassed 13 studies involving 980 patients (557 patients diagnosed with bacterial infections and 423 patients with non-bacterial infections). The overall occurrence rate of UTIs was determined to be 56.8%. The collective data showed a sensitivity of 82% (95% CI: 68%–90%) and a specificity of 86% (95% CI: 77%–91%). The positive and negative likelihood ratios were 5.66 (95% CI: 3.41–9.38) and 0.21 (95% CI: 0.12–0.40), respectively. However, the efficacy of sTREM-1 in predicting UTIs displayed a relatively low sensitivity of 18% (95% CI: 5%–51%). The authors postulated that, due to its limited sensitivity, sTREM-1 might not be an optimal biomarker for UTIs [24]. It was previously demonstrated that in pediatric patients diagnosed with acute febrile UTIs, plasma sTREM-1 levels were higher compared to healthy controls. Additionally, it was reported that sTREM-1 exhibited diagnostic performance with a sensitivity of 57% and a specificity of 50% [25]. In a study conducted by Sierra-Diaz et al., differences in TREM-1 expression in children with and without UTIs were investigated through flow cytometry. In their research, detectable TREM-1 expression was found in both groups via flow cytometry, with higher levels determined in those with UTIs [26]. Additionally, in their study, they investigated sTREM-1 levels in a subgroup using an ELISA kit, finding that the mean sTREM-1 level in children with UTIs was 140.6 ± 253 pg/dL. However, sTREM-1 levels were not evaluated in the children without UTIs. The authors reported that the higher TREM-1 levels in the UTI group could be associated with increased neutrophils and cytokine activity caused by bacteria [26]. The variation in the findings related to the predictive value of sTREM-1 in the aforementioned studies might stem from differences in the study populations (children or adults), the source of TREM-1 level measurements (whether from urine or blood), and the presence of diverse comorbidities in the participants of the studies.

This study revealed that the sTREM-1 levels of patients with ASP were elevated relative to those of the control group, and they markedly decreased in parallel with the clinical response following antibiotic therapy. This aligns with previous studies of various inflammatory diseases suggesting that sTREM-1 could be a significant biomarker for monitoring responses to antimicrobial therapy [9,22,27,28]. Furthermore, among these patients, there was a positive correlation between basal levels of sTREM-1 and the basal levels of other inflammatory markers. In a study conducted by Ehsanipor et al., it was demonstrated that sTREM-1 levels were higher in children with UTIs compared to the control group [14]. Their study showed that sTREM-1 exhibited a sensitivity of 83.3% and a specificity of 60% in identifying these patients. It was also reported that sTREM-1 levels did not differ between patients with lower and upper UTIs [14]. Another study of children with acute pyelonephritis indicated that elevated TREM-1 levels exhibited high positive predictive values [29]. In the current study, basal sTREM-1 levels demonstrated acceptable diagnostic performance in distinguishing patients with ASP. However, the contradictory outcomes from the limited studies in the literature indicate a need for more extensive research on sTREM-1 in UTIs.

This study has several significant limitations. First, this study was conducted at a single center and had a limited sample size. Another limitation of our study was that the sample was selected from patients undergoing emergency management for infections associated with ASP. Following the completion of antimicrobial therapies aimed at the urinary system infection, patients were referred to or consulted at the urology department for further management of kidney stones, which were potentially the source of infection, through extracorporeal shock wave lithotripsy (ESWL) or surgical interventions. Due to the prioritization of infection treatment and the subsequent referral for stone management, detailed data on the stone’s characteristics, such as stone location, number of stones, and size, were not systematically collected or analyzed as part of our study. Thirdly, the cytokines induced by sTREM-1 were not assessed in this study [30]. Additionally, the evaluation of leukocyte subtypes with flow cytometry analysis could shed light on the pathway through which sTREM-1 exerts its effects. Examining these factors in large-scale prospective studies might further clarify the role of sTREM-1 in cases of bacterial infection and non-bacterial inflammation.

## 5. Conclusions

Despite observations that levels of TREM-1 were higher upon hospital admission in patients with ASP and significantly decreased after treatment, the utility of sTREM-1 as a biomarker for predicting patients with ASP remains constrained when compared to established inflammatory markers. The decrease in sTREM-1 levels post-treatment, aligning closely with those of the control group, highlights its responsiveness to therapeutic interventions in ASP. Despite this, the median CRP levels remained elevated post-treatment, suggesting that sTREM-1, in conjunction with other biomarkers, could provide a more comprehensive overview of the infection status and the effectiveness of the treatment.

## Figures and Tables

**Table 1:** Demographic and clinical findings.

Variables	Control *n* = 23	ASP *n* = 46	*p*
Age, years	48.8 ± 12.7	47.7 ± 16.8	0.783
Male gender, *n* (%)	15 (65.2)	27 (58.7)	0.794
Comorbidity, *n* (%)	7 (30.4)	16 (34.8)	0.790
Hypertension	5 (21.7)	8 (17.4)	0.748
Diabetes mellitus	4 (17.4)	7 (15.2)	0.999
Urinalysis, *n* (%)			
Pyuria	-	46 (100.0)	<0.001 *
Positive nitrites	-	29 (63.0)	<0.001 *
Leukocyte esterase	-	42 (91.3)	<0.001 *
Positive uroculture ^‡^	-	28 (60.9)	<0.001 *
Radiological findings, *n* (%)			
Stone	-	46 (100.0)	<0.001 *
Hydronephrosis	-	9 (19.6)	<0.001 *
Hypoechogenic areas	-	3 (6.5)	<0.001 *
Thickening of pelvic wall	-	2 (4.3)	<0.001 *

Data are shown as mean ± SD or number and percentage (%). * *p* < 0.05 shows statistical significance. ASP, acute stone pyelonephritis. ^‡^ The patients had previously received their first doses of treatment in the emergency department.

**Table 2:** Comparison of laboratory findings for patients with acute stone pyelonephritis and the control group.

Variables	Control *n* = 23	ASP		*p* ^1^	*p* ^2^	∆*p*
		Pre-Treatment *n* = 46	Post-Treatment *n* = 46			
Leukocyte counts, ×10^3^/µL	7.2 (6.3–8.4)	12.4 (11.3–16.4)	7.6 (7.0–8.5)	<0.001 *	0.387	<0.001 *
CRP, mg/L	1.5 (0.8–2.5)	129.7 (77.8–230.0)	4.5 (3.4–8.9)	<0.001 *	<0.001 *	<0.001 *
Procalcitonin, μg/L	0.03 (0.02–0.04)	0.26 (0.10–2.60)	0.04 (0.03–0.08)	<0.001 *	0.080	<0.001 *
sTREM-1, pg/mL	68.4 (50.0–75.4)	98.6 (70.6–124.8)	65.7 (48.4–90.3)	<0.001 *	0.469	<0.001 *

Data are shown as mean ±SD or median (25th–75th quartile) or number and percentage (%). * *p* < 0.05 shows statistical significance. *p* ^1^, control vs. pre-treatment. *p* ^2^, control vs. post-treatment. ∆*p*, pre-treatment vs. post-treatment. ASP, acute stone pyelonephritis; CRP, C-reactive protein; sTREM-1, soluble triggering receptor expressed on myeloid cells.

**Table 3:** The relationship between sTREM-1 levels and other inflammatory parameters.

Variables	sTREM-1			
	Control		ASP	
	r	*p*	r	*p*
Pre-treatment				
Leukocyte counts	0.14	0.556	0.325	0.028 *
CRP	−0.297	0.204	0.412	0.004 *
Procalcitonin	−0.148	0.533	0.535	<0.001 *
Post-treatment change				
Leukocyte counts	-	-	0.279	0.132
CRP	-	-	0.265	0.157
Procalcitonin	-	-	0.311	0.036 *

* *p* < 0.05 shows statistical significance. ASP, acute stone pyelonephritis; CRP, C-reactive protein; sTREM-1, soluble triggering receptor expressed on myeloid cells.

## Data Availability

The data that support the findings of this study are available on request from the corresponding author. The data are not publicly available due to restrictions imposed by the ethics approval for personal raw data protection.
